# Concurrent Vascular and Pelvic Anomalies of the Kidney: A Rare Cadaveric Observation

**DOI:** 10.7759/cureus.102809

**Published:** 2026-02-02

**Authors:** Amartya Mishra, Vaishaali TM, Muhamed Faizal PA, Khursheed Raza, Ajay Patel, Ruchi Ratnesh, Neelam Kumari, Suyashi Sharma, Himel Mondal

**Affiliations:** 1 Anatomy, All India Institute of Medical Sciences, Deoghar, Deoghar, IND; 2 Anatomy, KMC Medical College, Maharajganj, IND; 3 Physiology, All India Institute of Medical Sciences, Deoghar, Deoghar, IND

**Keywords:** aberrant renal artery, cadaver, iatrogenic disease, iliac artery, kidney, kidney pelvis, renal artery, renal vein, trifid pelvis, vascular abnormalities

## Abstract

This case report describes the concurrent occurrence of rare renal vascular and pericalyceal variations and highlights their surgical significance. The renal vasculature and pelvis exhibit a wide spectrum of anatomical variations, many of which are clinically and surgically significant. Awareness of these deviations is of immense importance during retroperitoneal and renal surgeries and interventions. This report presents concurrent variations in both renal arteries and veins, as well as the renal pelvis, observed in a cadaver during routine dissection at the All India Institute of Medical Sciences, Deoghar, Jharkhand, India. Standard retroperitoneal dissection techniques were employed to expose and examine the structures. Observations were based on direct anatomical inspection and morphometric measurements. Variations were noted in the arterial supply, where at least three arteries supplied the right kidney - two arising from the abdominal aorta and one from the right common iliac artery - while two supplied the left kidney, arising from the abdominal aorta. These findings highlighted a deviation from the usual pattern of a single renal artery on each side. Bilateral venous variations were also observed. The left renal vein (LRV) had three tributaries: two venous channels forming the anterior limb and one draining into the inferior vena cava (IVC) as the posterior limb of a circumaortic venous ring. On the right side, three separate venous tributaries arising at different levels from the right kidney entered the IVC, along with an additional independent renal vein from the inferior renal pole draining inferiorly into the IVC. Furthermore, a trifid renal pelvis was found on the right side, characterized by three well-defined infundibula that drained separate calyces, resulting in a trifid configuration. No such variations in the pelvis were noted on the left side. This rare concurrence of venous, arterial, and pelvic variations underscores the importance of meticulous preoperative vascular mapping and radiological evaluation to prevent iatrogenic complications during nephrectomy, renal transplantation, vein catheterization, or other renal and retroperitoneal surgeries.

## Introduction

The kidney is supplied by a renal artery and drained by a renal vein, both of which show significant anatomical variations. Usually, a single renal artery arises from the abdominal aorta opposite the intervertebral disc between L1 and L2, while a renal vein drains into the inferior vena cava (IVC) [1]. According to Soares et al., one or more renal arteries may be present in 25-30% of individuals [2]. While isolated variations of renal artery variants are relatively common, there are very few documented cases of bilateral complex arterial configurations such as the crossed arterial pattern and differing branching types, which are relatively rare, highlighting the novelty of concurrent vascular deviations when studied anatomically [3].

Similarly, veins and the renal pelvis also show deviations from the usual configuration. The persistence or regression of embryonic vessels, along with rotation and ascent of the kidneys, gives rise to such variations [4]. These hold critical significance when viewed from a clinical and surgical perspective. Recognition of such variations in the vasculature and pelvis, especially when present in the same individual, is essential for accurate anatomical interpretation, effective surgeries, and prevention of intraoperative complications [5].

This case report describes concurrent variations observed during routine dissection, their clinical implications, and embryological basis, and provides future research recommendations.

## Case presentation

The dissection was performed on a formalin-embalmed 70-year-old female cadaver. The cadaver used in this study was donated to the Department of Anatomy, All India Institute of Medical Sciences, Deoghar, for educational and research purposes. The dissection was conducted in accordance with institutional ethical guidelines. Donor anonymity and dignity were strictly maintained. Variations were identified as deviations from standard anatomical descriptions. Multiple renal arteries or veins, anomalous origins or terminations, and atypical pelvicalyceal configurations were classified as variations. All structures were traced from origin to termination to confirm the findings.

A midline incision from the xiphoid process to the pubic symphysis provided access to the abdominal cavity. The anterior abdominal wall was reflected, and selected viscera were removed to expose the retroperitoneal structures. The peritoneum was stripped, and branches of the aorta between the superior mesenteric artery and the aortic bifurcation were traced [6] and finely dissected. Renal veins and their tributaries were traced from the renal hilum to their termination at the IVC. The renal pelvis and ureter were also traced from the hilum to the urinary bladder. Measurements were taken using a standard 150 mm (6”) digital caliper (AMAZINGs GmbH, Stuttgart, Germany) and a flexible measuring tape. All dissections and measurements were performed independently by two experienced observers. Each renal artery, vein, and pelvicalyceal structure was traced from origin to termination and examined carefully. While the observations were not conducted in a blinded manner, both observers cross-checked findings and repeated measurements to ensure accuracy and consistency. All measurements were taken from the inferior renal pole to the described structure along the external surface of the kidney using the digital vernier caliper.

**Figure 1:**
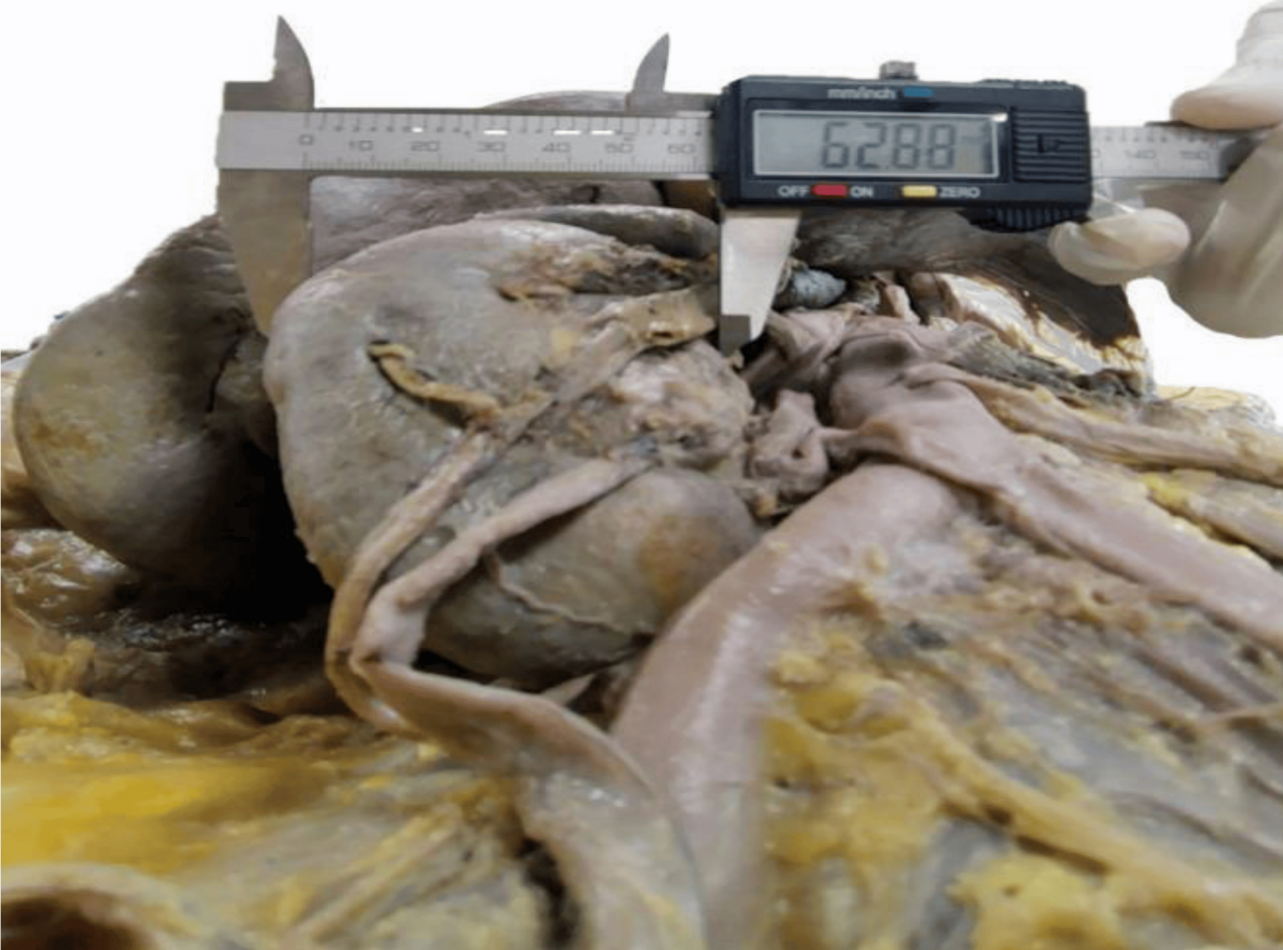
Measurement of the width of the right kidney at the level of the hilum

During dissection, the gross renal anatomy and measurements of both kidneys were recorded. Table 1 presents the dimensions of the right and left kidneys as measured by the digital vernier caliper. Figure 1 illustrates the measurement of the right kidney’s width at the hilum.

**Table 1:** Dimensions of the right and left kidney

Dimensions		Right kidney	Left kidney
Length		134.8 mm	135.2 mm
Breadth	At hilum	62.9 mm	69.2 mm
	At the upper pole	29.4 mm	32.8 mm
	At the inferior renal pole	34.1 mm	31.9 mm
Thickness		43.9 mm	47.2 mm
Length of the hilum		67.9 mm	71.3 mm

**Figure 2:**
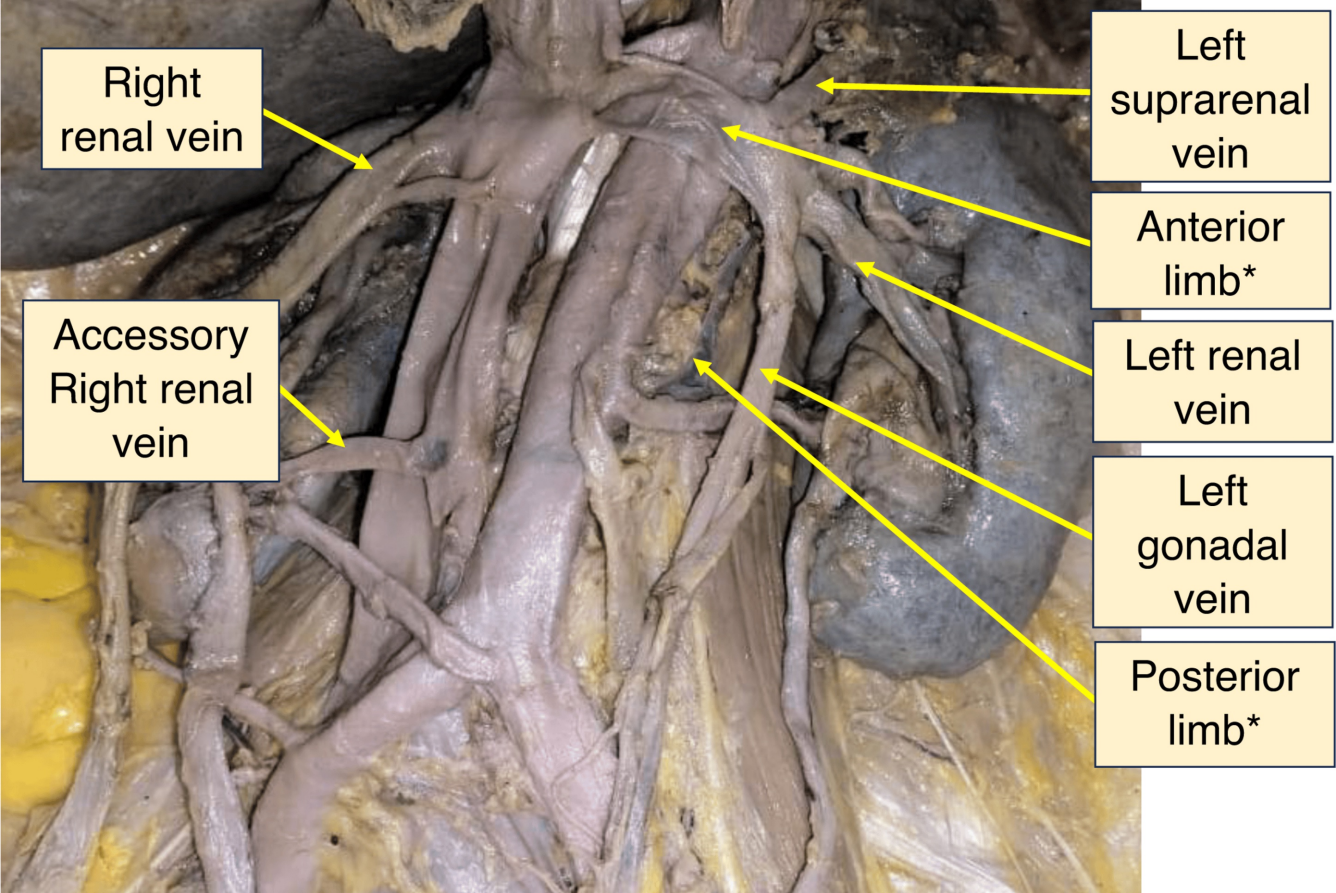
Venous drainage of both kidneys as seen in situ *of circumaortic renal collar

**Figure 3:**
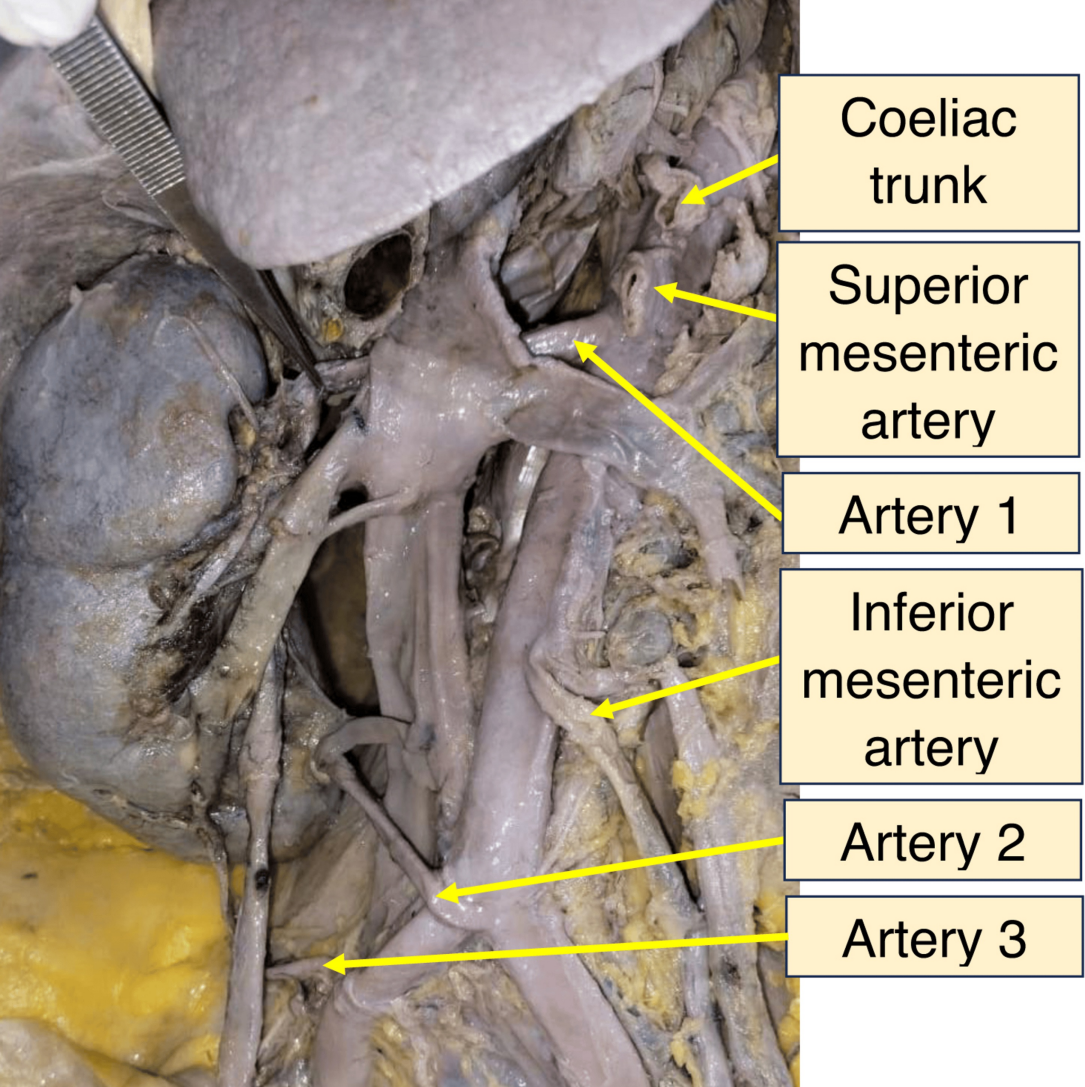
Photograph of the right side of the dissected abdomen showing multiple renal arteries as seen in situ (arteries 1, 2, 3)

Multiple renal arteries and veins supplying both kidneys were observed. Figure 2 shows the venous drainage of both kidneys in situ, while Figure 3 shows multiple right renal arteries (arteries 1, 2, 3) in situ.

**Figure 4:**
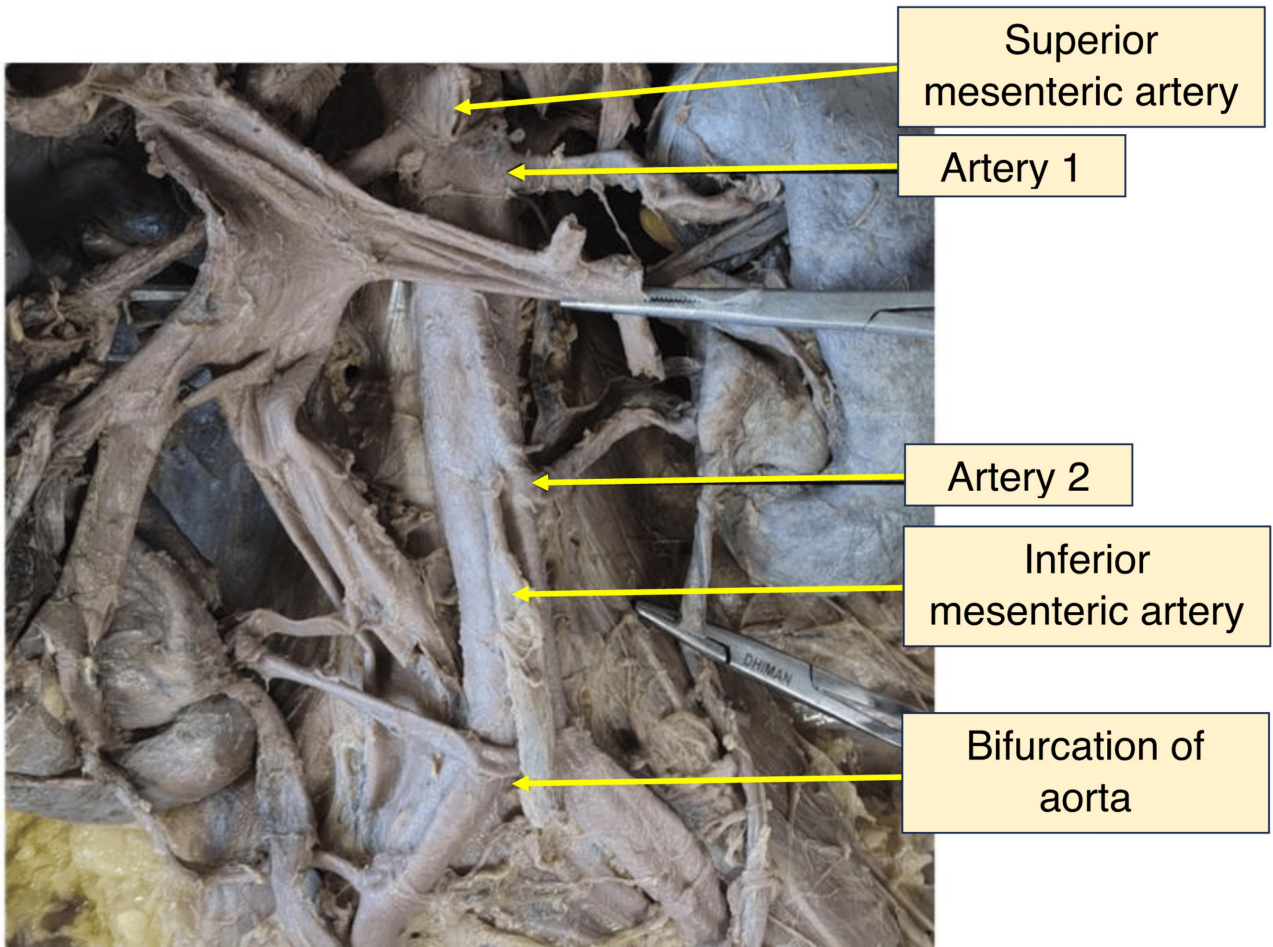
Photograph of the left side of the dissected abdomen showing multiple renal arteries as seen in situ (arteries 1, 2)

On the right, the highest renal artery originated laterally from the abdominal aorta just below the superior mesenteric artery and entered the hilum. The second artery arose abnormally at the aortic bifurcation and entered the inferior part of the medial margin. The third artery originated from the right common iliac artery and entered the inferior pole of the kidney. On the left, two arteries arose from the abdominal aorta at different levels proximal to its bifurcation and entered the hilum. Their specifications are shown in Figure 4 (arteries 1, 2). Measurements of the arteries to both kidneys are provided in Tables 2-3.

**Table 2:** Measurements of the arteries to the right kidney

Parameter		Artery 1	Artery 2	Artery 3
Origin		Just beneath the superior mesenteric artery (122.2 mm proximal to the bifurcation of the abdominal aorta)	At the bifurcation of the aorta	Right common iliac artery (42.23 mm from the bifurcation of the abdominal aorta)
Distance (from the inferior renal pole)		100.3 mm	26.1 mm	27.1 mm
Length (from aorta to entry point)		96.2 mm	71.2 mm	98.4 mm
Diameter	At origin	7.1 mm	4.9 mm	3.8 mm
	At the entry site	4.7 mm	3.7 mm	2.9 mm

**Table 3:** Measurements of the arteries to the left kidney

Parameter		Artery 1	Artery 2
Origin		123.5 mm proximal to the bifurcation of the aorta	64.2 mm proximal to the bifurcation of the aorta
Distance (from the inferior renal pole)		96.2 mm	55.1 mm
Length (from aorta to entry point)		61.2 mm	75.3 mm
Diameter	At origin	4.8 mm	5.4 mm
	At the entry site	3.9 mm	4.2 mm

The left renal vein (LRV) displayed a circumaortic variation with two distinct limbs: anterior (preaortic) and posterior (retroaortic). The anterior limb was formed from the union of two venous channels that drained the left kidney and received the left suprarenal and left gonadal vein, the former being proximal to the latter (concerning termination of LRV at IVC). The posterior limb (retroaortic) independently drained a portion of the kidney and joined the IVC posterior to the aorta. The anterior and posterior limbs originated from separate tributaries at the renal hilum, creating a venous collar around the abdominal aorta. The anterior limb measured 8.2 cm in length and originated approximately 8.2 cm from the inferior renal pole. The posterior limb was 10.53 cm long and originated about 5.3 cm from the inferior renal pole.

In the right kidney, the right renal vein (RRV) had three discrete tributaries. Vein 1 originated 9.1 cm from the inferior renal pole and was 2.3 cm long. Vein 2 was 2.8 cm long and originated 6.4 cm from the inferior renal pole. Vein 3 measured 3.5 cm and originated 3.5 cm from the inferior renal pole. An independent venous channel branched from the RRV and drained into the IVC proximal to the primary renal vein termination. Another channel, 4.4 cm long, drained separately into the IVC from a point 3.4 cm above the inferior renal pole. The right gonadal vein also drained into the RRV.

**Figure 5:**
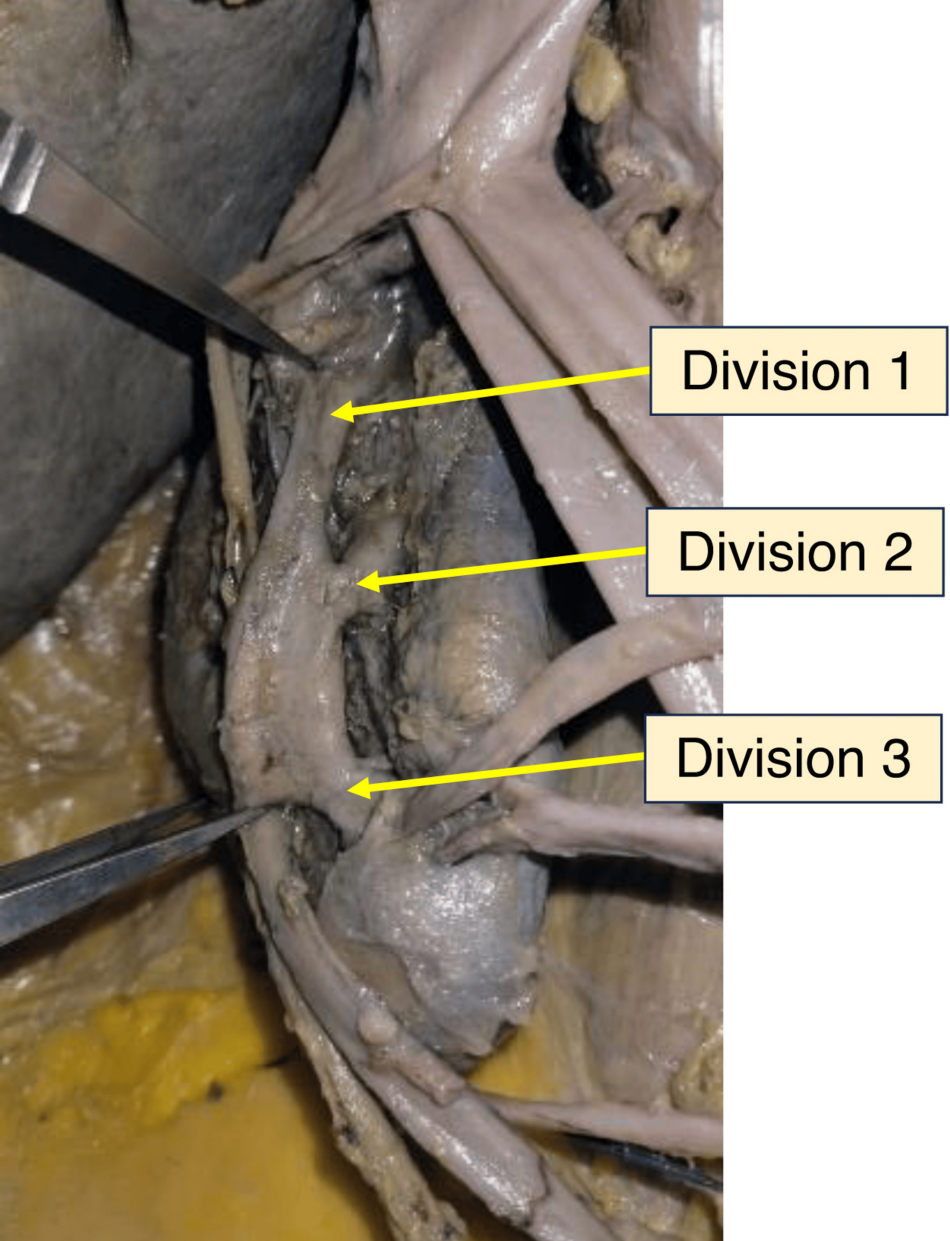
Renal pelvis reflected to show a trifid configuration

**Figure 6:**
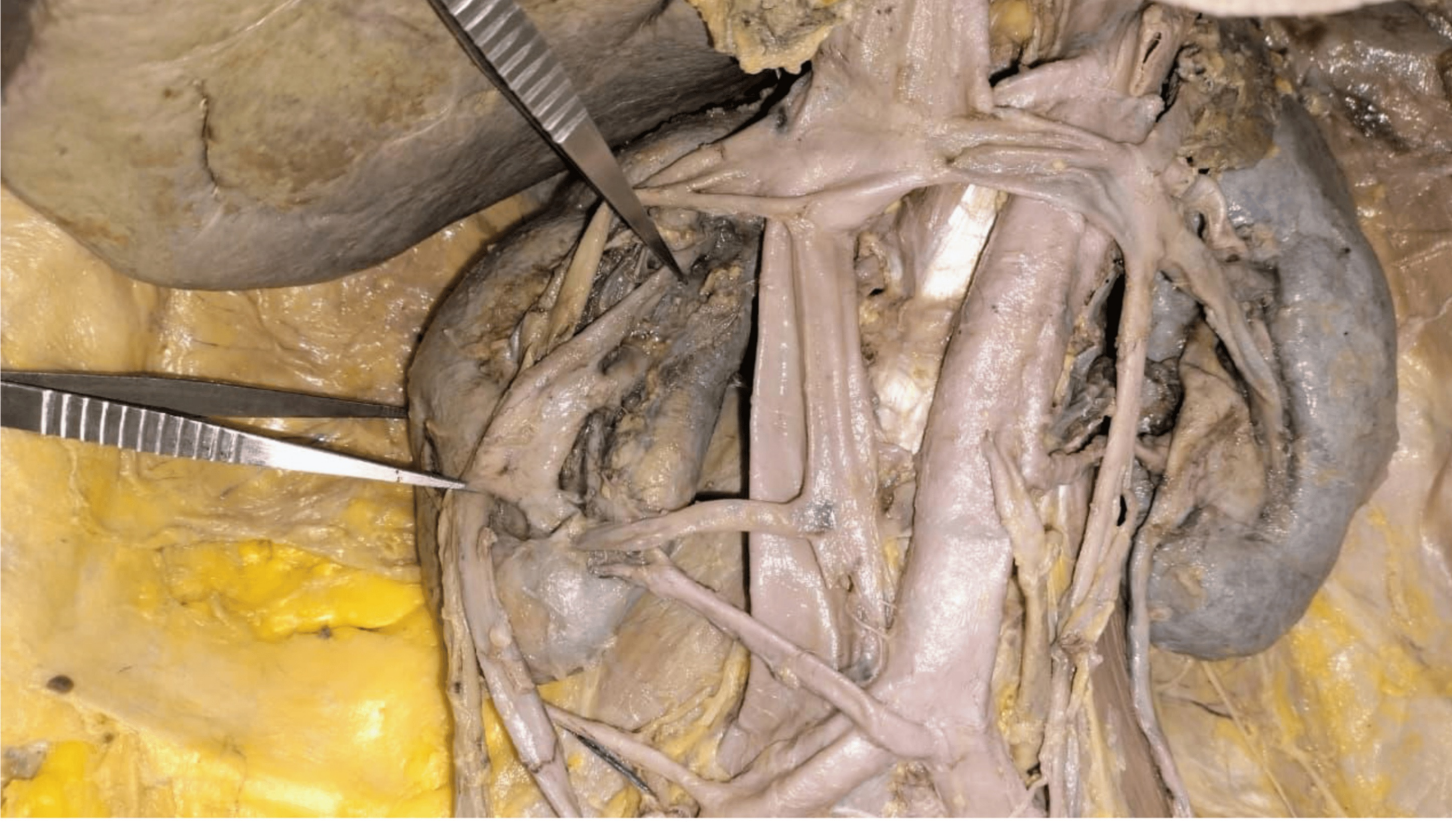
In situ dissection of the kidneys showing a trifid pelvis on the right, with no variations observed on the left

An unusual formation of the right renal pelvis was observed. The pelvis displayed a trifid arrangement, branching into three divisions, each directing flow to a separate major calyx. These divisions merged to form a single ureteric channel, as shown in Figures 5-6. Measurements of each division from the inferior pole of the kidney are provided in Table 4. The left kidney was drained by a single pelvis that continued into a single ureter. Measurements along the intrarenal collecting system were not performed.

**Table 4:** Measurements of each distinct division from the inferior pole of the kidney

Division of the renal pelvis	Distance from the inferior pole of the kidney
Division 1	10.4 cm
Division 2	6.5 cm
Division 3	3.5 cm

## Discussion

These findings are important as they demonstrate the coexisting anomalies in the vasculature and pelvis, rather than isolated anomalies, depicting the degree of synchronicity required during embryogenesis. There are previously reported cases of multiple renal arteries occurring in roughly 29% of kidneys [7], multiple renal veins in ~17% (more frequent on the right) [8], and a trifid renal pelvis is extremely rare and is reported only as isolated case reports [9], making the simultaneous occurrence of all three findings highly uncommon. In this cadaver, equally notable arterial counterparts accompanied the venous anomalies, including multiple renal arteries on both sides arising from the abdominal aorta and the right common iliac artery. Hostiuc et al. reported a prevalence of circumaortic LRV of 3.5% [8]. Additionally, the renal pelvis displayed a trifid configuration on the right - an extremely rare occurrence attributed to atypical branching of the ureteric bud during gestation [10]. Such variants are typically asymptomatic and are most often identified incidentally on imaging.

From a clinical perspective, the coexistence of multiple vascular and pelvicalyceal variations considerably heightens operative risk. The accessory renal artery, with an average external diameter of 3-4 mm, probably supplies a distinct renal segment. If inadvertently ligated during renal surgery, this could increase the risk of segmental ischemia. Both limbs of the circumaortic channel require careful identification, and failure to control them could result in significant hemorrhage. The trifid pelvis with three separate infundibula may complicate endourological procedures and predispose to stasis or incomplete drainage. Aberrant vessels complicate surgical dissection and vascular control, increasing the likelihood of severe hemorrhage or compromised renal venous drainage [11]. Ureteropelvic junction obstruction and subsequent hydronephrosis may result due to mechanical compression of the ureter by anteriorly crossing arteries [12]. In case of a circumaortic venous collar, during a nephrectomy, if both limbs are not clamped properly, it might lead to hemorrhage [13]. Inadvertent injury or ligation of these terminal arteries, lacking any collateral circulation, can precipitate ischemia within the dependent renal segment [14]. Such anomalies gain indispensable significance in procedures like renal transplantation, nephron-sparing surgeries, and abdominal aortic aneurysm repair [15]. Likewise, endourological or reconstructive approaches are highly influenced by pelvicalyceal variations, as unexpected branching becomes a crucial consideration in planning drainage or stenting, and may predispose patients to urinary stasis, recurrent infections, hydronephrosis, and calculi formation [16].

The mesonephric arteries, arising from the dorsal aorta, that fail to regress during the ascent of the kidney from the pelvis through the lumbar region, give rise to supernumerary arteries. According to a study, 30.76% of the supernumerary arteries arise from the aorta, while 12.82% from renal arteries [17]. Around the eighth week of intrauterine life, retroaortic and preaortic segments form a venous ring. Usually, the retroaortic ring regresses, but when both divisions persist, a circumaortic renal vein results [13]. The nephric duct gives rise to the ureteric bud, which undergoes sequential branching due to the effect of the metanephric mesenchyme to form the renal pelvis, calyces, and collecting ducts. These divisions, due to premature or excessive branching or incomplete fusion, may lead to the formation of a triplication or trifid pelvis, in which three infundibula separately drain separate calyces and join distally [18].

A major limitation of this case report is that it is cadaveric, and hence, the functional consequences of these vascular and pelvicalyceal variations could not be assessed. The embalming of the cadaver may have affected vessel diameter and tissue pliability, potentially altering spatial relationships. Also, the images included in this case report were photographed during routine dissection, and no calibrated imaging equipment was used, which would allow the addition of scale bars. Similarly, fixed directional indicators could not be reliably incorporated, as the relative position and orientation of the structures would vary during dissection and acquisition of the image. Furthermore, this is a single case and cannot be generalized to the population.

In the future, studies could combine radiological imaging and cadaveric data to better define the prevalence and anatomical variations of coexisting anomalies. Virtual three-dimensional reconstruction may assist in preoperative planning. Correlating such anatomical findings with surgical outcomes could improve patient safety during complex interventions.

## Conclusions

This case report highlights anatomical variations involving accessory arteries, anomalous venous channels, including a circumaortic channel, and a trifid pelvis, coexisting in the same individual. These variations increase intraoperative complexity and the risk of hemorrhage and urinary outflow obstruction if unrecognized. This report adds to the anatomical and surgical literature, emphasizing that renal variations should be dealt with as integrated patterns rather than isolated findings. Thorough preoperative imaging and assessment are therefore required to avoid complications.
